# Application of the Nano-Drug Delivery System in Treatment of Cardiovascular Diseases

**DOI:** 10.3389/fbioe.2019.00489

**Published:** 2020-01-31

**Authors:** Yudi Deng, Xudong Zhang, Haibin Shen, Qiangnan He, Zijian Wu, Wenzhen Liao, Miaomiao Yuan

**Affiliations:** ^1^The Eighth Affiliated Hospital, Sun Yat-sen University, Shenzhen, China; ^2^Guangdong Provincial Key Laboratory of Tropical Disease Research, Department of Nutrition and Food Hygiene, School of Public Health, Southern Medical University, Guangzhou, China

**Keywords:** nano-drug delivery system, cardiovascular disease, targeting strategy, application progress, safety

## Abstract

Cardiovascular diseases (CVDs) have become a serious threat to human life and health. Though many drugs acting via different mechanism of action are available in the market as conventional formulations for the treatment of CVDs, they are still far from satisfactory due to poor water solubility, low biological efficacy, non-targeting, and drug resistance. Nano-drug delivery systems (NDDSs) provide a new drug delivery method for the treatment of CVDs with the development of nanotechnology, demonstrating great advantages in solving the above problems. Nevertheless, there are some problems about NDDSs need to be addressed, such as cytotoxicity. In this review, the types and targeting strategies of NDDSs were summarized, and the new research progress in the diagnosis and therapy of CVDs in recent years was reviewed. Future prospective for nano-carriers in drug delivery for CVDs includes gene therapy, in order to provide more ideas for the improvement of cardiovascular drugs. In addition, its safety was also discussed in the review.

## Introduction

Cardiovascular diseases (CVDs) have become a serious worldwide public health problem, and the morbidity and mortality rank first above other diseases in the world (Gaurav et al., 2015). Faced with such a severe situation, developing drugs for the treatment of CVDs has become a top priority. Owing to the rapid development of nanoscience and outstanding performance of nanomaterials, nanotechnology has become a new solution to overcome the bottleneck of cardiovascular disease treatment. Nano-drug delivery systems (NDDSs) are a class of nanomaterials that have abilities to increase the stability and water solubility of drugs, prolong the cycle time, increase the uptake rate of target cells or tissues, and reduce enzyme degradation, thereby improve the safety and effectiveness of drugs (Quan et al., 2015; Gupta et al., 2019). NDDSs can be administered by various routes including inhalation, oral administration, or intravenous injection, remaining better bioavailability. In recent years, more scholars have started to develop nano-drug carrier system for the diagnosis and therapy of CVDs.

Additionally, as the application of nanomaterials increases, the exposure hazard of nanomaterials in clinical application also raises, resulting in the consequence that nanomaterials will have more opportunities to interact with blood vessels, blood, and their components, which will have an important impact on the human health. Therefore, this article mainly introduced the different types of NDDSs, their targeting strategies and application in CVDs, and the safety of nanomaterials was discussed as well.

## Types of the NDDSs

**Figure 1:**
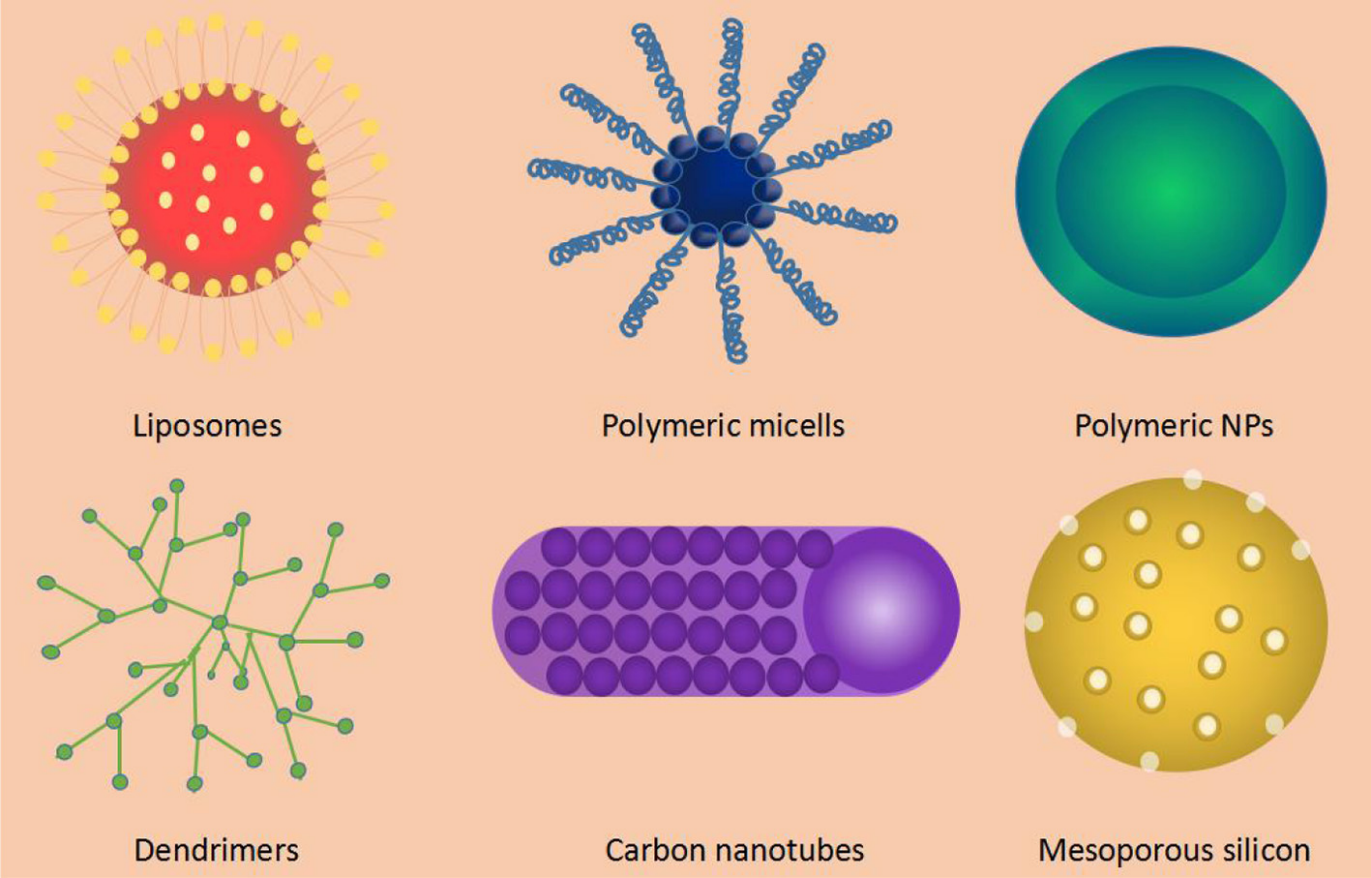
Common types of nano-drug carriers.

NDDSs refer to material in which at least one dimension is in the range of nanometer scale (1–100 nm) or composed of them as basic units in three-dimensional space (Cooke and Atkins, 2016; Zhou et al., 2018). As an effective means to optimize the drug delivery, NDDSs have become a research hotspot in the field of pharmacy and modern biomedicine (Matoba et al., 2017). The investigation of NDDSs has been for more than 40 years, creating a mass of nano-drug carriers. According to the composition of the materials, the nanomaterials used in NDDSs can be divided into organic, inorganic and composite materials. The following is a description of several common NDDSs and their features (Figure 1, Table 1).

**Table 1:** Category and features of nano-drug carriers.

**Category**	**Structure**	**Drug loading**	**Advantages**	**Limitations**	**References**
Liposomes	Lipid bilayer	Physical encapsulation/Chemical connection	Great biocompatibility, none immunogenicity	Low stability, easy leakage of hydrophilic drug	Jain and Jain, 2018; Yue and Dai, 2018
Polymeric nanoparticles	Nanospheres/Nanocapsules/Polymer-based nanoparticles with lipophilic core		Good stability, low leakage of drugs	Intravenous toxicity	Elsabahy and Wooley, 2012; Hu et al., 2018
Polymeric micelles	Core/shell architecture formed by self-assembly		Easily prepare, increase stability of hydrophobic drug	Low stability, depolymerization after dilution	Cagel et al., 2017
Metal nanomaterials	Nanoparticles, nanorods, nanocapsules, nanocuboid, and nanowire		Antibacterial properties, magneto-optical response characteristics	Toxicity, hard to degrade	Vimbela et al., 2017
Inorganic non-metallic nanomaterials	The same size with a adjustable pore size		Stable size, large surface area and pore volume, high drug loading	Extremely slow biodegradation rate	Yu F. et al., 2018

### Liposomes

In general, liposomes are lipid vesicles formed by ordered phospholipid bilayer with cell-like structure (Landesman-Milo et al., 2013). As a type of drug carrier, liposomes show many advantages, such as non-toxic, non-immunogenicity, sustained-release drugs, prolonging drug action time, changing drug distribution *in vivo*, improving drug treatment index, reducing drug side effects, and so on (Yingchoncharoen et al., 2016). Liposomes can not only be easily developed for the entrapment of hydrophilic and ionic molecules, but compatible with hydrophobic drug (Chandrasekaran and King, 2014). Hydrophobic drugs can be surrounded by the bimolecular structure of phospholipids, and hydrophilic drugs, especially those containing genes, can be attached to the hydrophilic region of liposomes. The particle size, potential, and surface chemistry can be adjusted by modification of different lipid materials. Among various type of liposomes, cationic liposomes are positively charged, indicating that they may result in dose-dependent cytotoxicity and inflammatory responses, and as a kind of complexes, they may interact non-specifically with negatively charged serum proteins. Neutral lipids (Chapoy-Villanueva et al., 2015) and pH sensitive liposomes (Fan et al., 2017) are two ways to solve the above problems.

### Polymer Micellar Co-delivery System

Polymer nanoparticles, another carriers for the delivery of drug, can be classified into non-biodegradable materials and biodegradable materials (Shi et al., 2019a,b). The synthetic polymer materials mainly include poly(lactic-co-glycolic acid) (PLGA), polyvinyl imine (PEI), polycaprolactone (PCL), polyvinyl alcohol (PVA), and so on (Danhier et al., 2012; Wei et al., 2018). These polymers exhibit biocompatibility, non-toxicity and no teratogenicity. Its degradation products, including oligomerization and final products, have no toxic effect on cells, and can coexist stably with most drugs. Natural polymers are mainly categorized into polysaccharides, peptides (Li et al., 2012), Chol and cyclodextrin inclusion complexes. Polymer nanoparticles usually formed by self-assembly of Amphiphilic block copolymers, are stable in the core and can be used to intercept insoluble drugs (Afsharzadeh et al., 2018). The stable structure of polymer nanoparticles is beneficial to the uniformity of particle size and the controlled release of drugs (Wang et al., 2011), and can effectively overcome the influence of gastrointestinal environment during oral administration. Their nanoscale and large surface area are conducive to uptake of drugs in cells and better bioavailability. Unfortunately, some polymer nanoparticles, have some drawbacks. For example, Chitosan, a natural polymer, is incompatible with biologic fluids, which can lead to particle degradation and reduce the working efficiency. Structural changes can be taken to solve its deficiency. Combining chitosan with polyethylene glycol, the conjugate has a unique endocytosis and macrophage phagocytosis mechanism (Yang et al., 2017). In addition, the modification of chitosan with a polypeptide can improve its working efficiency (Ping et al., 2017).

### Dendritic Macromolecules

Macromolecules are synthetic, various-shaped and usually branched. Macromolecules shaped as sphere can be arranged in monodisperse space and mostly used as nano-carriers to be used for the administration and dissolution of insoluble targeted drugs. Dendritic macromolecules with unique branch structure, are also monodispersion and their molecular weight can be controlled. Besides, a large number of ready-made surface functional groups and hydrophobic environment are exist in the packaging, which make them an excellent drug delivery material (Kesharwani et al., 2012). Because of their excellent biological properties, dendritic macromolecules are widely used in biomedical and pharmaceutical fields, but the existence of surface cationic charge also limits their clinical application.

### Metal Nanomaterials

The most commonly used metal nanomaterials are gold and silver nanomaterials, shaped in different structures that can be divided into/like nanoparticles, nanorods, nanocapsules, nanocuboid, and nanowire (Baeza et al., 2017). In addition to being used as nano-contrast agent for CT and surface-enhanced Raman spectroscopy, gold nanomaterials are also used in photothermal treatment of tumors and rheumatoid arthritis. As many studies shown, the application fields of silver nanomaterials mainly involved antibacterial, anti-infection and anti-tumor (Pietro et al., 2016). Moreover, some therapeutic drugs can be physically loaded into hollow gold or silver nanostructures (Liang et al., 2014), or chemically bonded to the surface of nanoparticles to achieve targeted delivery of the drugs. However, the removal of gold nanomaterials in human body is too slow, and the toxicity of silver ions *in vivo* limits the application of these metal nanomaterials in the treatment of chronic diseases.

### Inorganic Non-metallic Nanomaterials

Inorganic non-metallic nanomaterials mainly include quantum dots, iron oxide, silicon, grapheme, and so on (Khafaji et al., 2019). Quantum dots (QDs), that is, semiconductor nanocrystals, are particularly focused on fluorescence imaging because of their unique luminous properties, while iron oxide nanoparticles are chiefly lay on the study of new MRI contrast agents (Jayagopal et al., 2009; Hauser et al., 2016; Su et al., 2017; Wei H. et al., 2017). Among them, mesoporous silicon nanomaterials have attracted more and more attention in the therapy of diseases in recent years due to its large surface area and porous structure (Wang W. et al., 2016). Those Inorganic nanomaterials can be used to improve the transport efficiency of drugs and genes in mammal cells through the integration of different functional groups. Meanwhile, they are suggested to be a kind of joint carrier with development potential. However, the bio-safety of inorganic non-metallic nanomaterials would be a considerable obstacle to their application in clinic (Perioli et al., 2019).

### Composite Nanomaterials

In addition to the above nanomaterials, the preparation of composite nanomaterials with different properties is also under exploration in many studies. For example, metal or inorganic non-metallic nanomaterials are introduced into polymer or lipid nanomaterials to prepare multifunctional NDDSs containing both therapeutic drugs and contrast agents. Metal and inorganic nanomaterials are decorated or modified by organic materials to improve their physical and chemical properties, *in vivo* kinetic behavior and biocompatibility; and some NDDSs with special structure and diversified functions can be prepared by the combination of different metals and inorganic materials.

## Targeting Strategy of the NDDSs

The targeted design of NDDSs focuses on the diagnosis and therapy of cancer in the early stages of development, but recent researches argued that lesion cells or tissues of CVDs can also be targeted, even easier to targeted than tumor tissues with multiple physiological barriers. Compared with conventional preparations, the metabolic time of nano-transporter drugs in the blood circulation may be prolonged. By regulating pH value (Gao et al., 2018; Yi et al., 2018), temperature (Wei L. et al., 2017), light (Ding et al., 2011), ultrasound or biological enzyme (Zhang et al., 2019), the rate of those targeted nano-transporter drugs can be controlled to function longer.

### Passive Target Transfer

#### Enhanced Vascular Permeability

**Figure 2:**
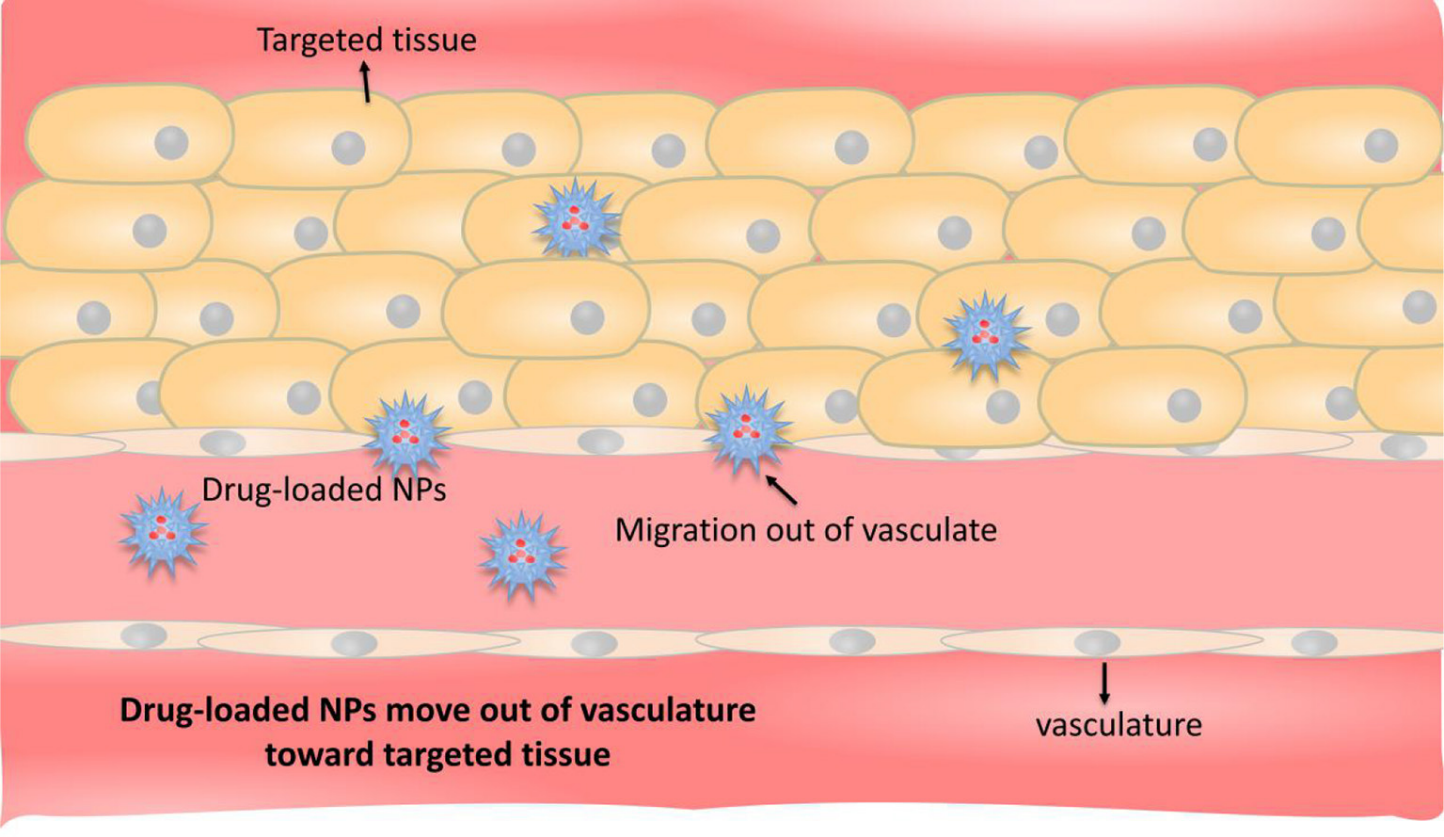
Schematic representation of passive targeting. The occurrence and development of CVDs are chronic inflammatory processes in which vascular permeability is usually increased, and nanoparticles of appropriate size pass directly through the blood vessels and release the drug at the site of the disease.

Passive targeted transport mainly utilizes high permeability and high retention (EPR) effects (Figure 2) (Holback and Yeo, 2011). EPR refers to the fact that some molecules or particles tend to accumulate in tumor tissues (Dinarvand et al., 2011). The microvascular endothelial cell space in normal tissue is dense and intact, and NDDSs loaded with drug, generally in high molecular weight, are not easy to pass through the vascular wall. The tumor tissue is rich in blood vessels and poor in structural integrity (Torchilin, 2011). Those drug-loaded NDDSs in high molecular weight can selectively pass through the vascular wall and remain in the tumor tissue. A large number of studies have shown that nano-drug carriers with particle size <100 nm can be located and targeted to solid tumor tissues by EPR. Compared with the direct administration method, the nano-drug carrier can increase the accumulation of the drug in the tumor tissue by more than 10 times, greatly improving the bioavailability (Maeda et al., 2013). But it is discovered that EPR effect can also be used in various CVDs, not only for tumors. In some course of CVDs, for example, the occurrence and development of AS is a chronic inflammatory process, where vascular permeability is often increased, which is very similar to that of solid tumors. Vascular endothelial permeability provides an effective means for NDDSs to deliver from the lumen side to the interior of the plaque. The nano-drug carriers entering the circulation are also ingested by inflammatory cells (monocytes or macrophages), and these drug-carrying cells migrate to plaque inflammation, allowing drugs to be delivered in another way (Flogel et al., 2008).

Due to the size and surface characteristics of a portion of nanomaterials, they are rapidly cleared in the blood during intravenous injection, making nanomaterials unsuitable for drugs that require long cycle times. In this case, nano-coating technology can be applied to the nano-system for certain concealment, and the rate of administration of the coating agent can be also controlled and adjusted. This technology is particularly suitable for NDDSs in the treatment of CVDs. Developers on NDDSs have employed poly (ethylene glycol) (PEG) in particle design. In fact, PEG is a flexible hydrophilic polymer that can form a hydrated layer when grafted onto the surface, effectively reducing the adsorption of proteins on the surface (Jokerst et al., 2011). The tissue plasminogen activator is encapsulated in the nanoparticles, making the nanosystem concealed in some degree, thus protecting the tissue plasminogen activator from inactivation by plasma inhibitors and prolonging the half-life (Hemmati and Ghaemy, 2016).

#### Shear-Induced Targeting

Studies have shown that as the intima grew outward (toward the lumen) in CVDs, such as advanced AS or myocardial infarction, thrombosis or microthrombus occurs, stenosis of the blood vessels follows and blood flow rate through the plaque increases, and thus the fluid shear force increases. The mean blood fluid shear force in the normal vasculature is <70 dyne·cm^−2^, while the blood fluid shear force in the AS plaque stenosis is up to 1,000 dyne·cm^−2^ (Korin et al., 2012). Therefore, the design of blood fluid shear-sensitive nanoparticles can achieve physicochemical targeting by utilizing the difference of blood fluid shear force between AS plaque and normal blood vessels. Holme et al. (2012) prepared a lenticular lipid nanoparticle vesicle with two sides convex. The drug-loaded nanometer can maintain structural stability in normal blood vessels, and the configuration change can be utilized to release the drug under the action of high blood fluid shear force through the blood circulation to the AS plaque. Inspired by the activation of platelets under the action of local high blood fluid shear forces in AS plaques and adhesion to plaque blood vessels, the researchers constructed a nanoparticle aggregate that can be assembled locally in plaques (Korin et al., 2012). First, the authors prepared PLGA nanoparticles with a particle size of about 180 nm and entrapped tissue plasminogen activator, and then obtained a PLGA nanoparticle aggregate with a particle size of 3.8 nm through spray drying. When the nanoparticles were exposed to the local high fluid shear stress of the AS plaque, they could be decomposed into 180 nm PLGA nanoparticles, and relied on the strong penetrability of the small particle size nanoparticles to enter the local thrombus of the plaque. The thrombolytic effect maximized the efficacy, significantly reduced the dose required for thrombolysis and the side effects of thrombolysis. In ischemic cardiomyopathy, the endothelial gap in ischemic myocardium widened, thus altering the shear of blood flow, and the concentration of polysaccharide from Ophiopogon japonicus in ischemic myocardium was twice as high as that in normal rats (Lin et al., 2010). Tan et al. found that both shear stress and blood flow shear rate of vascular wall could affect the aggregation of nanoparticles (Tan et al., 2011).

#### Magnetically Guided

Magnetically guided nanoparticle is an interesting “pseudo-passive” targeting method. Theoretically, the application of an external magnetic field can direct magnetic nanoparticles to the disease site (Prijic and Sersa, 2011). Recent evidence suggests that this strategy is beneficial for CVDs (Chandramouli et al., 2015). Alam et al. (2015) compared the effects of several nano drug carriers on atherosclerotic plaque imaging. Those Nanoparticles include iron oxide particles, superparamagnetic iron oxide nanoparticles, ultra-small superparamagnetic iron oxide nano-carrier, and very small superparamagnetic iron oxide nanoparticles. The results showed that the ultra-small superparamagnetic iron oxide nanoparticles have better vascular wall penetration ability and plaque retention than other groups. Some researchers have pointed out that the external magnetic field helps to transport particles from the cell-free layer which lacks red blood cells to the vessel wall (Freund and Shapiro, 2012).

### Active Targeted Transhipment

**Figure 3:**
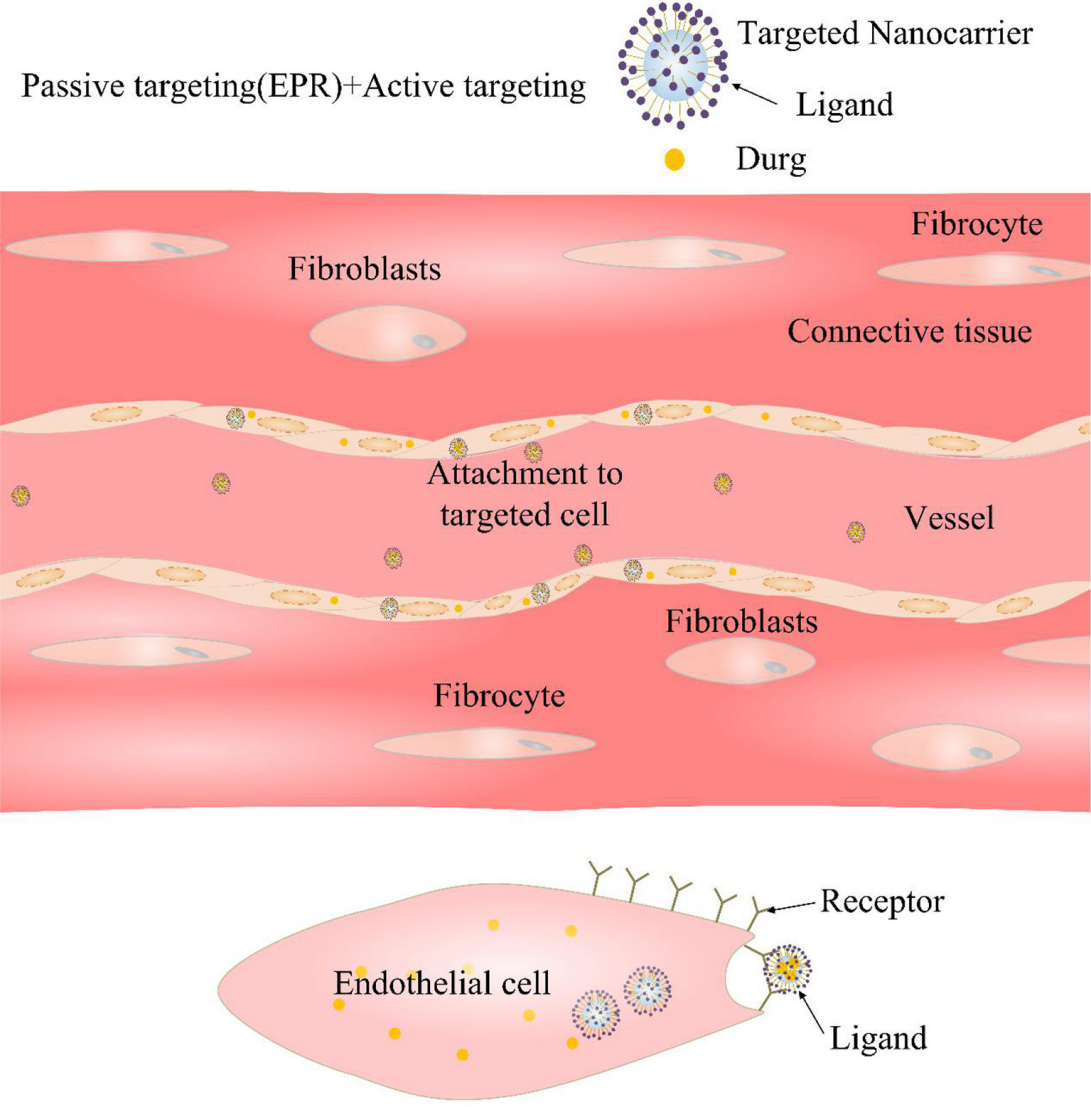
Diagrammatic sketch of active targeting. The surface of the nano-carrier is grafted with a targeting ligand, which is strongly bound to the selective cell surface by ligand-receptor binding.

On the basis of passive targeting, using the special pathological features of CVDs to develop an active targeting strategy for CVDs can improve the targeted delivery efficiency of drugs to the lesions of CVDs, which aroused researchers strong interest. Active targeting is primarily directed to functional modification of NDDSs with one or more targets to allow the drug to reach a particular site (Figure 3) (Matoba and Egashira, 2014). That is to say, introducing a functional group or active substance that specifically interacts with diseased tissues or cells into the surface of the nano-drug carrier will enhance carriers targeting (Lee et al., 2006; Gullotti and Yeo, 2009). Some active targets are discussed in detail below (Table 2).

**Table 2:** Some molecules which can provide the active target for NDDSs.

**Superfamily**	**Lectin adhesion molecules**		**Ig-superfamily cell adhesion molecules**			**Hyaluronic acid receptor**
**Cell adhesion molecule**	**P-selectin**	**E-selectin**	**VCAM-1**	**ICAM-1**	**PECAM-1**	
CD classification	CD62P	CD62E	CD106	CD54	CD31	CD44
Surface expression	Inducible	Inducible	Inducible	Constitutive and up-regulated upon induction	Constitutive	Widely distributed, cell surface, transmembrane
Temporal expression	Expression is fast and transient; internalized within 20 min	Peak expression at 4 h (*in vitro*); declines to baseline within 24 h during inflammation	Very low copies/cell; increases to 10^4^-10^5^ copies/cell	10^4^-10^5^ copies/cell in normal tissue *d*; 3- to 5-fold increase in inflammation	10^6^ copies/cell	–
Ligands	Leukocyte expressing sialyl-Lewis X	Leukocyte expressing sialyl-Lewis X	Leukocyte with β1 integrin VLA-4 (α4β1) and α4β7	Leukocyte with β2 integrins (e.g., LFA-1 and Mac-1)	Leukocyte with β1 and β3 integrins Heparin proteoglycans	Hyaluronic acid
Function	Leukocyte tethering and rolling		Leukocyte firm adhesion		Leukocytes transmigration; angiogenesis	Participation in heterogeneous adhesion

#### Active Targeting of Vascular Endothelial Cells

At different stages of CVDs, vascular endothelial cells are in an inflammatory activation state. Compared with normal vascular endothelial cells, some small molecules including intercellular adhesion molecule-1 (ICAM-1), vascular adhesion molecule-1 (VCAM-1), integrins, selectins, and so on, are often overexpressed, which provides the active target for NDDSs (Glass and Witztum, 2001). It is showed that the conjugation of lung-targeted single-stranded variable fragment/liposome together with platelet endothelial cell adhesion molecule-1 (PECAM-1) antibody increases liposome transport to the pulmonary vascular system and strengthen its anti-inflammatory effects (Hood et al., 2018). In 2013, Yang et al. decorated the surface of silica nanoparticles with anti-VCAM-1 monoclonal antibody. The nanoparticles were able to bind to sites of inflammation before they were taken up by endothelial cells (Yang et al., 2013).

Based on the pathological features of high expression of ICAM-1 in early vascular endothelial cells of AS, Paulis et al. (2012) modified the antibody anti-ICAM-1 which actively targets ICAM-1 on the surface of liposomes and used it to load contrast agents (gadolinium). Studies have shown that the liposome could achieve the activated targeting of vascular endothelial cells and AS plaques through the specific action of anti-ICAM-1 and ICAM-1. However, competitive binding of circulating white blood cells to the ICAM-1 site and blood flow shearing could reduce the targeting function of liposomes to AS plaques. The authors optimized the binding degree of liposome to ICAM-1 by screening liposome particle size, antibody and lipid ratio, and obtained higher active targeting efficiency.

E-selectin is a surface glycoprotein of endothelial cells, which can promote the attachment of monocytes/macrophages and lymphocytes to induce inflammatory response, and eventually cause the occurrence and development of CVDs, such as AS (Ma et al., 2016). E-selectin can also be used as a target for nano-transport drugs. Functional liposomes carrying mouse H18/7 mAb (specific antibody to E-selectin) were used to act on interleukin (IL)-1β-activated human umbilical vein endothelial cells and non-interleukin (IL)-1β-activated human umbilical cord Vein endothelial cells. It was found that the ability of functional liposomes to target activated human umbilical vein endothelial cells is 275 times that of the non-activated type (Flaht-Zabost et al., 2014).

AT1 rises in myocardial when myocardial infarction or heart failure happened. Dvir et al. (2011) designed a polyethylene glycol liposomes (142 ± 8 nm), that could carry therapeutic payloads (such as growth factors, cytokines, etc.) and released them in a controlled manner. The ligand attached on these liposomes is a string of amino chain sequenced Gly-Asp-Arg-Val-Tyr-Ile-His-Pro-Phe (binding sequence of AT1 receptor), which could direct the nanoparticles to the infarction heart.

#### Active Targeting of Macrophages or Foam Cells

Macrophages or foam cells play a key role in the development of AS. In the early stage of AS, mononuclear/macrophages were recruited to activate vascular endothelial cells, and overexpressed some inflammation-related receptor molecules in an inflammatory environment, such as CD44 and interleukin-4 (IL-4) receptors, etc. Imaging and drug delivery for macrophages or foam cells using NDDSs will facilitate monitoring of disease progression and drug treatment in AS.

For example, Lee et al. (2015) linked 5β-cholic acid and fluorescent dye Cy5.5 to the carboxyl group of the HA skeleton by chemical bonding and formed nanoparticles (HA-NPs) by self-assembly. Compared with nanoparticles (HGC-NPs) constructed with chitosan backbones that did not target CD44 receptors, HA-NP could significantly increase the uptake of activated macrophages, and the plaque site of ApoE^−/−^ mouse (AS model) was more targeted. Fluorescence co-localization studies indicated HA -NP was mainly distributed in macrophages in plaques.

Park et al. (2008) used phage library screening technology to optimize the amphiphilicity of the target IL-4 receptor peptide (CRKRLDRNC) which was modified on amphiphilic chitosan (with ethylene glycol chitosan as the backbone and 5β-cholate bonded) by chemical bond. Then nanoparticles with the function of targeted macrophages in AS plaque are obtained in a self-assembled method.

#### Targeting Vascular Basement Membrane Collagen

It has been reported that the vascular basement membrane of damaged blood vessels and inflammation sites is rich in collagen IV (Col IV) (Duner et al., 2015). In 2013, Kamaly et al. (2013) ligated the 7 amino acid oligopeptide molecule KLWVLPK (PLEA-β-PEG-Col IV) targeting collagen IV at the PEG end of the PLGA-β-PEG block copolymer and used it to package Act-26 (With anti-inflammatory and inhibition of leukocyte extravasation), thus nanoparticles (Ac2-26 Col IV NPs) targeting damaged blood vessels and collagen sites of inflammation sites were prepared. The results showed that Act-26 Col IV NPs reduced the migration and adhesion of neutrophils to the inflammation site and inhibited the development of inflammation. Further, in 2016, some researchers prepared nanoparticles (Col-IV IL-10 NPs) containing anti-inflammatory factor IL-10 by self-assembly using PLGA-p-PEG-Col IV and PDLA-PEG-OMe targeting collagen LV (Kamaly et al., 2016). After intravenous administration of Ldlr^−/−^ mice, it was found that Col-IV IL-10 NP significantly increased the content of IL-10 in the plaque, and had better AS treatment effect than free IL-10.

In addition, multi-target nano-carriers with multiple inflammatory cell characteristics have been studied. PLNs incorporated these often ignored biophysical design criteria of platelet-mimetic discoid morphology and flexibility, then integrated these design parameters with the platelet-mimetic biochemical heteromultivalent interactive functions by dendritic presentation of multiple peptides that bind simultaneously to both activated natural platelets and injured endothelial sites (Anselmo et al., 2014).

Whether it is passive targeting or active targeting, the final targeting efficiency depends on the biological and physical properties of nanoparticles. The biological and physical properties includes particle size and distribution, targeting unit types, surface chemistry, morphology and density (Morachis et al., 2012). For the body, the development stage, type as well as location of CVDs and tumor, vascular wall shear rate, blood composition and its fluid type, together with other factors will greatly affect the targeting efficiency (Charoenphol et al., 2011). Although the application of active targeting NDDSs in clinical diagnosis and therapy is extremely attractive, its development is still facing great challenges. Those challenges are mainly reflected in two aspects: one is the limitation of the discovery of ideal target; the other is that there are still many bottleneck problems in the design and preparation of effective targeting nanosystem.

### Multifunctional Responsiveness NDDSs

Multifunctional responsive NDDSs is a kind of drug carrier with better targeting ability, which is developed on the basis of the above two targeting modes of nano-drug carrier. In addition to having the previous targeting ability, this kind of carrier is generally composed of stimulating responsive materials, which can be released under the stimulation of the special environment of the focus site, thus reducing the release in the normal tissue and increasing the drug accumulation of the lesion tissue. At the same time, diagnostic molecules can be assembled or labeled on nano-carriers to compose an integrated diagnosis and therapy system.

## Application of the NDDSs in the Diagnosis of CVDs

**Figure 4:**
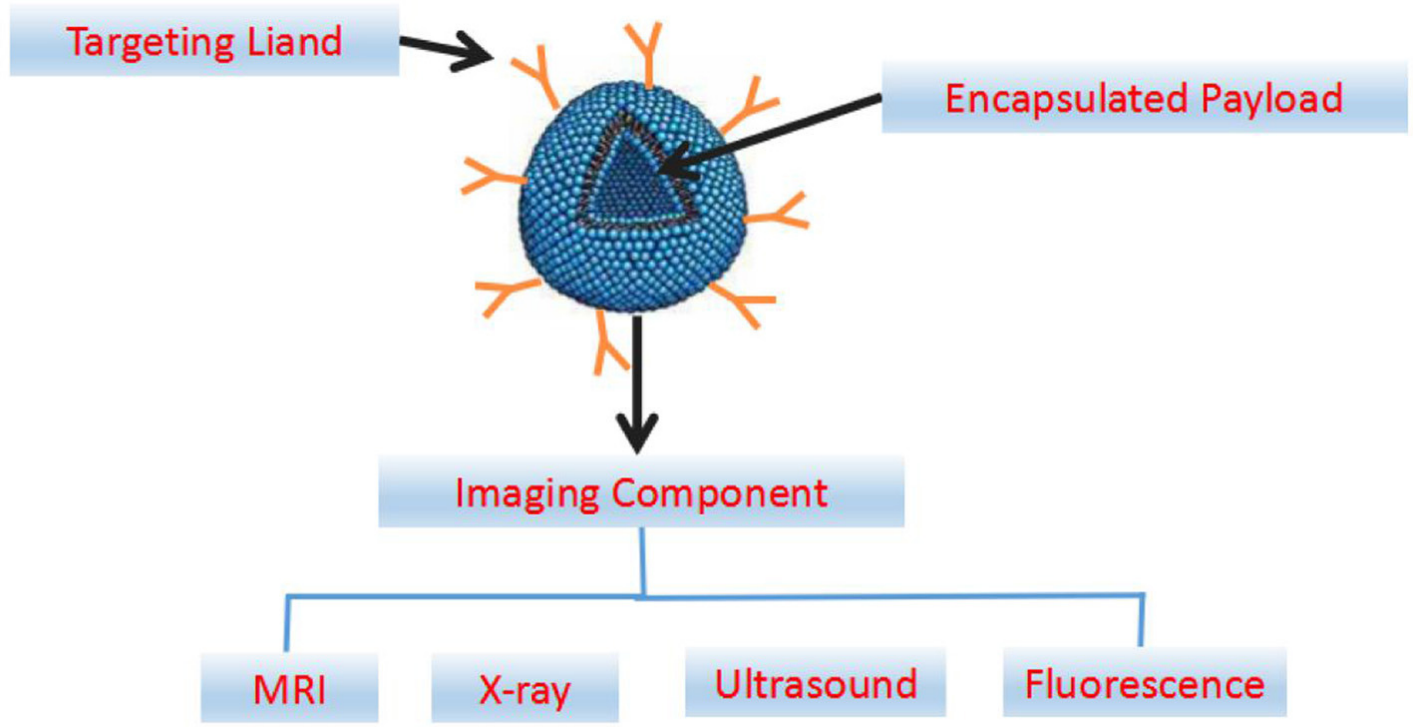
Abridged general view map of targeted nanoparticles engineered for imaging and drug delivery. The components of a multifunctional nanocarrier include a ligand for cellular targeting, and an encapsulated payload for delivery of the therapeutic agents. The imaging components can be incorporated in the interior payload, on the targeting ligand or associated with the nanoparticle shell.

Early, rapid and accurate detection is important for effective prevention and treatment of CVDs. The application of molecular imaging in the diagnosis of CVDs has been paid more and more attention in recent years. In addition to the constant innovation of various imaging technologies, new contrast agents are the key to real-time, fast, high sensitivity and high resolution diagnostics. Compared with conventional contrast agents, nano-contrast agents have the following advantages: (1) *in vivo* stabilization, regulable distribution, and prolonging the half-life of contrast agents or drugs; (2) controllable physical and chemical properties (such as chemical composition, size) and imaging performance; (3) specific identification of certain biomolecules; (4) ability of multimodal imaging realization; (5) values in individualized diagnosis and therapy are expected to be realized (Attia et al., 2016). By designing specific nano-probes with the unique chemical signal molecules of diseased tissues determined by pathological studies, the contrast agent can be directed to the lesion area in the early stage of the disease for magnetic resonance imaging (MRI), X-ray imaging, fluorescence imaging, and contrast-enhanced ultrasound (US) imaging (Figure 4).

### Magnetic Resonance Imaging

In many imaging methods, magnetic resonance imaging is non-invasive, safe, and high resolution, and it is good for soft tissue imaging. However, the sensitivity of MRI is not high (10^−3^-10^−9^ M). The complexes of gadolinium commonly used in clinical practice are used as T1-weighted imaging contrast agents, and gadolinium has certain nephrotoxicity. Fe_3_O_4_ nanoparticles are considered to be non-toxic T2-weighted imaging contrast agents (Corot et al., 2006). Compared with tinctures, they have high sensitivity, good tissue compatibility and superparamagnetism (Kim et al., 2007). Targeted contrast agents are used to accumulate MRI probes at a sufficiently high concentration (in micrograms to milligrams) in the target tissue to achieve a high signal to noise ratio.

It is discovered that vascular imaging can be performed in the early stage of cardiovascular disease formation, and drugs can be administered for treatment after the magnetic nanoparticles are injected into the body. Yoo et al. (2016) loaded the hydrophilic lipid (amphiphilic) gadolinium chelating agent diethylenetriamine pentaacetic acid (DTPA) into a dendritic polymer and then wrapped it in the kernel of amphiphilic micelles and connected with fibrin binding agent. Thus, its targeting to atherosclerotic plaque was enhance, and can be used for early detection of thrombus. Winter et al. chose paramagnetic nanoparticles targeting integrin αvβ3 to inject intravenously into high fat fed New Zealand white rabbits to detect neovascularization in plaques in the early stage of AS (Winter et al., 2008).

### X-Ray Imaging

Imaging with radionuclides plays a crucial role in the field of nuclear medicine (Mottu et al., 1999, 2002). Radionuclides are not only sensitive but also quantifiable. Positron emission tomography (PET) and single photon emission computed tomography (SPECT) are the most common types (Alie et al., 2015). At present, radionuclide-labeled nanomaterials can be used to monitor the embolization process and the distribution of nanomedicine to achieve targeted imaging (Mottu et al., 2002; Okamura et al., 2002; Torchilin, 2002; James et al., 2006). For example, the researchers used ^186^Re-BMEDA (Bao et al., 2003) and 99^m^Tc-PEGylate-labeled (Bao et al., 2004) doxorubicin liposomes to perform SPECT, which can trace the distribution of drugs in the body, and also promote drug release. The nanoparticles can be used to detect the formation of atherosclerotic plaques by CT and to judge the prognosis as well. Galperin et al. injected iodine nanoparticles contrast agent (N1177) into mice via vein. It was found that the contrast agent gathered in macrophage rich tissue, and the signal of atheromatous plaques could be significantly enhanced, and the enhancement time could last for more than 30 min (Galperin et al., 2007). In 2016, Chhour et al. (2016), used 11 mercaptoundecanoic acid (11-MUDA) to encapsulate gold nanoparticles, found that gold nanoparticles could accumulate in foam cells of atherosclerotic plaques and increase the contrast of imaging.

### Fluorescence Imaging

Optical imaging is a powerful imaging method with the advantages of no radiation, no invasion, high resolution and good controllability, but its penetration is poor. Fluorescence imaging is usually performed by using fluorescein to generate fluorescence signals. Near-infrared fluorescence (NIRF) probes are widely used because of their strong penetrating power and safety. They have been used in small animal living imaging systems and clinical tumor transformation. At present, a large number of nano-drug carriers, such as liposomes, metal, or non-metallic nanoparticles can enclose NIRF to achieve optical imaging of blood vessels (Weissleder and Ntziachristos, 2003; Setua et al., 2010; Sevick-Muraca, 2012). Its application in cardiovascular disease imaging has been paid more and more attention. McCarthy et al. bound the group of near infrared light activated therapeutic (NILAT) with macrophage-targeted magnetic nanoparticles(MNP) and prepared a kind of diagnostic and therapeutic nanoparticles (McCarthy et al., 2006). The experiment results shown that within 24 h of administration, the nanoparticles were reached in area. Wang Y. et al. (2016) injected profilin-1 magnetic iron oxide nanoparticles (PF1-Cy5.5-DMSA-Fe_3_O_4_-NPs) focusing on profilin-1 into the vein of atherosclerotic mice. It was found that the magnetic iron oxide nanoparticles were aggregated in carotid atherosclerotic plaques. There was a good correlation between the MRI signal of the animals injected with PC-NPs and the fluorescence intensity of NIRF imaging *in vitro*.

### Ultrasound Imaging

Compared with fluorescence imaging, ultrasound imaging has natural advantages in medical imaging including safe, convenient, and real-time. Nano-ultrasound imaging materials that can be targeted to vascular-related markers have been developed. For example, vascular ultrasound nanoparticles that can be targeted to high expression of the vascular endothelial growth factor receptor 2 (VEGFR2) not only provide a more clear ultrasound imaging of tumor blood vessels, but also promote drug localization in blood vessels (Rojas et al., 2018). Marsh et al. had developed perfluorocarbon nanoparticles targeting blood fibrin, carrying the thrombus drug streptokinase for the diagnosis and therapy in thrombus (Marsh et al., 2007). The drug-loaded particles are synthesized by evaporation/dispersion technique with a diameter of about 250 nm and can be used for ultrasonic imaging.

### Multi-Modal Bioimaging

At present, multi-modal imaging technology using a combination of different types of imaging methods can integrate different imaging methods to produce synergistic effects, providing more comprehensive and accurate image information for accurate diagnosis and precise treatment of CVDs. For instance, it was found that ^64^Cu-labeled SPIO-loaded doxorubicin nanoparticles could be used for MRI and PET (Yang et al., 2011). It has been reported that Cy5, sputum, and folic acid can be embedded in gold nanoparticles to achieve trimodal optical imaging, MRI and CT imaging in mice (Chen et al., 2016). This multimodal imaging and integration of diagnosis and treatment will be a new direction for the development of cardiovascular nanomedicine in the future.

## Application of the NDDSs in the Treatment of CVDs

### The NDDSs in AS

AS is the most common type of CVDs, often leading to a stroke or heart attack. The formation of AS begins with endothelial dysfunction. Plaque-induced coronary artery stenosis can cause ischemic cardiomyopathy, while plaque rupture can cause acute myocardial infarction (Nabel and Braunwald, 2012; Wall, 2013). Mechanisms of plaque instability include enhanced vascular permeability, Platelet endothelial cell adhesion molecules (PECAM) expression, macrophage aggregation, and expression of proteases, which can be targets for intervention. The drug can be delivered to atherosclerotic plaques by nano-drug carrier, to effectively prolong the half-life of drug plasma, increase the concentration of lesions and reduce side effects. The treatment strategies of these nano-drug carriers including regulating lipoprotein level, reducing the degree of inflammation, inhibiting of neovascularization, preventing coagulation, and so on (Table 3). These treatment strategies are used as interventions to development of AS, reduce plaque area or stabilize vulnerable plaques (Chetprayoon et al., 2015; Bejarano et al., 2018).

**Table 3:** Application of the NDDSs in the AS.

**Carrier**	**Ligand**	**Drug**	**Intervention mode**	**Model**	**References**
Liposome	–	Glucocorticoids (PLP)	Intravenous injection (I.V.)	Rabbit	Lobatto et al., 2010
Dendrimeric nanoparticles	Mannose	Liver-x-receptor ligands(LXR-L)T091317	Intravenous injection (I.V.)	LDLR^−/−^ mouse	He et al., 2018
Acetylated β-CD materials (Ac-bCDs)	–	Rapamycin (RAP)	Subcutaneous injection	ApoE^−/−^ mouse	Dou et al., 2016
Polylactic acid-glycolic acid (PLGA)	Hyaluronan (HA), apolipoproteins A-1 (apoA-1)	Simvastatin	*In vitro*	Dynamic system of Endothelial macrophage Co-culture	Zhang et al., 2017
Peptide amphiphilic nanofiber	A1 apolipoprotein	Drug liver X receptor agonist GW 3965(LXR)	Intravenous injection (I.V.)	Mouse	Mansukhani et al., 2019
Acetal-CD (Ac-bCD) and ROS-sensitive CD-CD (Ox-bCD)	Acetaldehyde, sensitive to ROS	Rapamycin	Intraperitoneal injection (I.P.)	Mouse	Dou et al., 2017

### The NDDSs in Hypertension

At present, many kinds of drugs are applied for the treatment of hypertension, including angiotensin converting enzyme inhibitors, vascular angiotensin antagonists, central sympathetic nerve drugs, adrenergic receptor blockers, diuretics and vasodilators (Sharma et al., 2016). However, all these antihypertensive therapeutic drugs have obvious defects, including short plasma half-life, low bioavailability, toxic and side effects (upper respiratory tract abstraction, angioedema, reflex tachycardia, extreme hypotensive effect, and so on) (Alam et al., 2017; Martin et al., 2017; Niaz et al., 2017). Conversely, nano-drug carriers can provide prominent advantages mentioned above (Table 4) (Kimura et al., 2009). Some researchers have made olmesartan into a nanoemulsion system. Compared with the conventional dose, the nanoemulsion group has better blood pressure lowering effect, longer maintenance time, and can produce nearly three times the dose reduction (Alam et al., 2017).

**Table 4:** Application of the NDDSs in the treatment of hypertension.

**Carrier**	**Drug**	**Intervention mode**	**Model**	**References**
Poly (D, L-lactide) (PLA)	Aliskiren	Gavage	Male spontaneously hypertensive rats (SHR)	Pechanova et al., 2019
Niosomes	Lacidipine (LAC)	Oral	Hypertensive rats	Qumbar et al., 2017
Lliposome	Valsartan	Cutaneous penetration	Experimental hypertensive rats	Ahad et al., 2016
Chitosan (CS) polymer	Captopril, amlodipine and valsartan	Oral	–	Niaz et al., 2016
Chitosan and polyethylene glycol composite sol.	Nitric oxide, NO precursor (nitrite)	Oral	–	Cabrales et al., 2010

### The NDDSs in Pulmonary Hypertension

Pulmonary hypertension, a progressive highly dangerous disease, is characterized by increased pulmonary vascular resistance and elevated pulmonary artery pressure. Prostaglandin I, Endothelin receptor antagonist, type 5 phosphodiesterase inhibitor, etc. are common vasodilators for pulmonary hypertension. These vasodilators have shown some effects, but the overall therapeutic ability is limited. For solving this problem, nano-mediated drug delivery system has gradually become an important alternative strategy (Table 5). Bosentan is a selective and competitive Endothelin receptor antagonist, which is loaded into nanoparticles and has a solubility of seven times as much as that of unprocessed bosentan (Ghasemian et al., 2016).

**Table 5:** Application of the NDDSs in pulmonary hypertension.

**Carrier**	**Ligand**	**Drug**	**Intervention mode**	**Model**	**References**
Nanostructured lipid carriers (NLCs)	–	Sildenafil (SC)	Endotracheal administration	A549 cells, rat	Nafee et al., 2018
Polymeric nanoparticles	Vitamin A	Nitric oxide(NO)	–	Hepatic stellate cells (HSCs)	Duong et al., 2015
Nanocomposite particle (nCmP)	–	Tacrolimus (TAC)	Direct intervention	A549 cells	Wang Z. et al., 2016
Liposome	Peptide CARSKNKDC (CAR)	Fasudil and superoxide dismutase (SOD)	Direct intervention, endotracheal administration	Pulmonary endothelial and smooth muscle cells, rat	Gupta et al., 2015
Poly(D,L-lactide-co-glycolide) nanoparticles	–	Silaenafil	Endotracheal administration	–	Beck-Broichsitter et al., 2012
Liposome	–	Cerivastatin	Endotracheal administration	Rat	Lee et al., 2018

### The NDDSs in Myocardial Infarction

Reperfusion is mainly used in the early stage of myocardial infarction, but it can cause apoptosis, calcium overload and reactive oxygen species. These factors cause the opening of the mitochondrial membrane permeability transition pore (MPTP) and the increase of mitochondrial outer membrane permeability, thereby promoting cardiomyocyte apoptosis and necrosis (Hausenloy and Yellon, 2013). Clinically, the drug therapy for myocardial ischemia mainly depends on growth factors, cytokines and some small molecular compounds. These drugs have the same disadvantages of the above traditional drugs. The high permeability of blood vessels and enrichment of monocytes in ischemic myocardium can be harnessed to deliver drugs by targeting ability of nano-drug carriers (Table 6).

**Table 6:** Application of the NDDSs in myocardial infarction.

**Carrier**	**Ligand**	**Drug**	**Intervention mode**	**Model**	**References**
Poly(D,L-lactide-co-glycolide) (PLGA)	–	Insulin-like growth factor (IGF)-1	Injection in the heart	Mouse	Chang et al., 2013
Low molecular weight polyethyleneimine	Deoxycholic acid (PEI1.8-DA)	siRNA against Src homology region 2 domain-containing tyrosine phosphatase-1 (SHP-1)	Cardiac administration	Myocardial infarction (MI) rats	Dongkyu et al., 2013
Liposome	P-selectin	Vascular endothelial growth factor (VEGF)	–	Myocardial infarction (MI) rats	Scott et al., 2009
Distearyl phosphatidylethanolamine polyethylene glycol	Atrial natriuretic peptide (ANP)	Oleate adenosine prodrug (Ade-OA)	Intravenous injection (I.V.)	Acute myocardial infarction (AMI) rats	Yu J. et al., 2018
Polylactic co-glycolic acid nanoparticles	–	Vascular endothelial growth factor (VEGF)	Injection into the peri-infarct region	Infarcted mouse	Oduk et al., 2018
Lipid core nanoparticles (LDE)	–	Methotrexate (MTX)	Intraperitoneal injection(I.V.)	Myocardial infarction (MI) rats	Maranhao et al., 2017

### The NDDSs in Other CVDs

As a new drug delivery platform, nano-drug delivery system also performs well in other CVDs. Coronary artery allogeneic angiopathy is an inflammatory proliferation process that undermines the long-term success of heart transplantation. Lipid nanoparticles coated with methotrexate or paclitaxel were injected intravenously into rabbits which fed cholesterol-rich diet and received an ectopic heart transplant, both of which reduced macrophage infiltration in the graft (Barbieri et al., 2017). Myocardial ischemia is mainly due to the decrease of aortic perfusion in the heart, resulting in insufficient oxygen supply and unstable myocardial energy metabolism, thus forming a pathological state that cannot support the normal work of the heart. Liposomes coated with phenytoin (PHT, a non-selective VGSC inhibitor) were prepared by thin film dispersion. The results showed that PHT-encapsulated liposomes partially inhibited I/R injury-induced CD43^+^ inflammatory monocyte expansion and reduced infarct size and left ventricular fibrosis after intravenous injection of the rat myocardial I/R injury model (Zhou et al., 2013).

Vascular restenosis is the process of stenosis and obstruction after the interventional treatment of the blood vessels, such as angioplasty, arteriotomy, implantation of an endovascular stent, and so on (Wang et al., 2018). Some scientists (Banai et al., 2005; Kamath et al., 2006; Nakano et al., 2009; Schröder et al., 2018; Xi et al., 2018) have proposed that in the site of angioplasty, catheter-intervention techniques are used to infuse the drug-loaded nanoparticles into the injury site, enabling angioplasty, and topical administration in one step. The nanoparticles can enter the arterial wall through the damaged endothelium, localize, reside in and between cells, and then slowly release the drug (Wu et al., 2019). Therefore, the lesion vessel can be maintained at a relatively high concentration for a long period of time, which is beneficial to fully exerting the drug effect, and finally effectively prevents and treats vascular restenosis.

## Application of the Co-loaded Nano-System in the CVDs

Drug combination therapy (including genes) is the treatment of two or more drugs to patients at the same time. In clinical practice, this therapy has been widely used for disease treatment. The purpose of this combination therapy is often due to the synergistic effect between drugs, or the therapeutic effect of multiple drugs is greater than that of a single drug. In recent years, many co-loaded nano-systems have been developed to carry common drugs and/or genes, especially siRNA to treat CVDs.

### Application of RNAi in the Treatment of CVDs

RNA interference (RNAi) is a gene-specific silencing mechanism present in eukaryotic cells and an important measure for resisting foreign genes and infections during biological evolution. RNAi was first discovered in *Caenorhabditis elegans* (Braukmann et al., 2017), then in 2001, it was demonstrated to occur in mammalian cells (Lendeckel et al., 2001). RNA interference includes micro RNA (miRNA), small interfering RNA (siRNA), Piwi-interacting RNA, and long non-coding (lncRNA). RNAi technology, also known as gene silencing, introduces double-stranded RNA (dsRNA) consisting of sense and antisense RNAs corresponding to a certain mRNA sequence into cells, degrading mRNA homologously complementary thereto, and inhibiting the expression of cell-specific genes. The rapid development of RNAi research has driven it from experimental technology to therapeutic development tools (Katyayani et al., 2017), and RNAi has potential value in the treatment of CVDs (Kwekkeboom et al., 2014; Tadin-Strapps et al., 2015; Hoelscher et al., 2017). At the same time, RNA interference therapy also has challenges in the treatment of CVDs, including the toxicity, targeting, time-effect, and effective delivery system of RNA, which limits its widespread use in the clinic and is urgently needed to be solved and improved (Cotten et al., 1992; Sioud, 2015; Kasner et al., 2016; Navickas et al., 2016; Zhou et al., 2016). Table 7 indicated the future direction of cardiovascular RNA interference.

**Table 7:** Future directions in cardiovascular RNA interference.

**Investigation**	**Novel strategies**	**Future perspectives**	**References**
RNAi imaging *in vivo*	Prolyl hydroxylase domain protein 2 (PHD2)- short interfering RNA (shRNA) sequence followed by a hypoxia response element-containing promoter driving a firefly luciferase reporter gene	Imaging of RNAi distribution in space and time in experimental animal models	Huang and Wu, 2011
Cardiac-targeted RNAi	Cardiotropic adeno-associated virus 9 (AAV9)-based silencing of Ca^2+^ cycle regulator phospholamban for the treatment of severe heart failure via intravenous route	Cardiac-specific gene knockout for therapeutic purposes, and as an alternative for cardiac-specific inducible knockout models	Suckau et al., 2009
Induction of alloimmune tolerance by RNAI	In heart transplantation models, RNAi induced alloimmune tolerance through silencing of toll-like receptor (TLR) adaptors My D88 and TIR-domain-containing adapter-inducing interferon-β (TRIF)	*In vivo* treatment of recipients with siRNAs to My D88 and TRIF prolonged allograft survival	Zhang et al., 2012
Plaque stabilization by RNAI	Lentivirus-based RNAi to silence chymase increased plaque stability in *in vivo*	Chymase as a target for plaque stabilization in vasculature as an RNAi target	Guo et al., 2013
Monocyte-targeted RNAi	Nanoparticle-encapsulated synthetic siRNA for silencing of monocytic chemokine receptor C-C chemokine receptor type 2 (CCR2) in myocardial infarction	Non-viral RNAi delivery system targeting monocytes *in vivo*, with high translational potential	Majmudar et al., 2013

### Co-loaded Gene and Drug Nano-System

For overcoming the problems in the delivery process and realizing the broad potential of RNAi-based therapeutics, safe and efficient nano delivery systems are needed. The apolipoprotein B (ApoB) siRNA was encapsulated into the liposome vector. After 48 h, the ApoB mRNA of the macaque liver decreased, and the maximum silencing rate exceeded 90%. ApoB protein, serum cholesterol, and low-density lipoprotein levels began to decrease 24 h after treatment and continued until day 11 (Zimmermann et al., 2006). Some researchers have used chitosan nanoparticles to construct and package small interfering RNA (siRNA) against PDGF-B mRNA expression vector, and then transfected into vascular smooth muscle cells (vSMC) of rabbit arterial wall damaged by balloon catheter, using therapeutic ultrasound for gene delivery. The results showed that the nanoparticles significantly inhibited the expressions of PCNA and PDGF-B mRNA in intimal vSMCs while the local intimal thickness and area were also reduced remarkably (Xia et al., 2013). Nox2-NADPH expression is significantly increased in the infarcted myocardium. Somasuntharam et al. (2013) demonstrated acid-degradable polyketal particles for Nox2-siRNA to the post-MI heart, which not only reduced siRNA degradation, but also inflammation.

Some pharmaceutical companies have developed new nano-dosages that deliver siRNA to the right cells at the right time (Hayden, 2014). Healthy volunteers (serum LDL levels of 3 mmol/L or higher) were injected intravenously with ALN-PCS or placebo developed by Alnylam Pharmaceuticals (Fitzgerald et al., 2014). ALN-PCS is a siRNA that inhibits the synthesis of PCSK9 and is assembled in lipid nanoparticles. PCSK9 protein in the human body cycle was reduced 70%, and LDL was reduced by 40% after intravenous injection of ALN-PCS.

**Figure 5:**
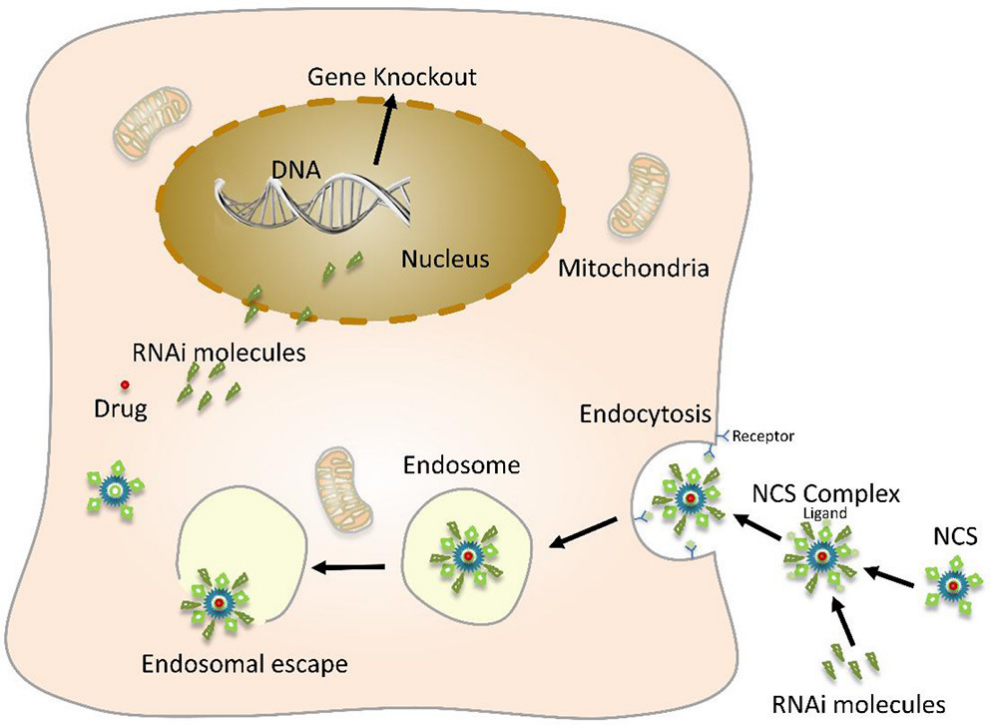
Schematic diagram of nanoparticles-mediated gene and drug delivery. Nano-carrier system (NCS) encapsulates or adsorbs a drug, a therapeutic foreign gene molecule on its surface, and also couples a specific targeting molecule to the surface of the nano-carrier and then binds to a cell surface-specific receptor through a targeting molecule. This enables safe and effective targeted genes and drug treatment.

The co-loaded gene and drug nano-system combined with nanotechnology and gene interference technology, the packaged substances have a synergistic effect, and the therapeutic effect is much better than the single treatment (Figure 5). Carvedilol, a kind of anti-hypertrophic drug that simultaneously blocks β-adrenergic receptors non-specifically in various organs, is widely used and effective. The non-specific genome-wide downregulation of p53 expression by specific siRNA efficiently abrogates cardiac hypertrophy. However, it can cause extensive tumorigenesis affecting bystander organs. Rana et al. (2015) encapsulated these bioactive molecules with stearic acid modified carboxymethyl chitosan (CMC) nanopolymers conjugated to a homing peptide for delivery *in vivo* to hypertrophied cardiomyocytes, resulted in effective regression of cardiac hypertrophy.

## Safety of the NDDSs

As the researches of nanomaterials go further, an increasing number of nanomaterials are prepared as NDDSs, but the unclear toxicity and the lack of systematically study of materials themselves restrict their further application. When the particle size enters the nanometer scale, it will show strong surface effect, small scale effect, quantum scale effect and macroscopic quantum tunneling effect (Gatoo et al., 2014).

Relatively few studies discuss about toxicity of NDDSs, particular in cardiovascular toxicity. But the tissue of cardiovascular system is considered to be the key site of NDDSs induced toxicity, which can produce great impact on the disease prognosis. Studies have revealed that nanomaterials could enter the blood circulation through respiratory tract, digestive tract, skin and other mucous membranes, and inevitably interacted with the blood system, immune system and other organs or tissues including plasma proteins and immune proteins, blood cells and immune cells, and so on.

The safety evaluation of the NDDSs is mainly focused on the toxicological study of the health effect. At present, the cardiovascular toxicity of nanomaterials based on animal and cell level shown that the toxicity was closely related to a series of undesirable effects induced by nanomaterials, including oxidative stress, inflammation apoptosis, blood aggregation and cardiac signal transduction (Donnini et al., 2000; Savic et al., 2003; Chen and Von, 2005; Qinghua et al., 2005). Among them, inflammatory reaction and oxidative stress are recognized as the main mechanisms of cardiovascular toxicity of nanomaterials.

Inflammatory response can affect the occurrence and development of CVDs including hypertension, myocarditis, AS, acute myocardial infarction and heart failure. Some researchers have found that if nano-carriers have not been removed in time, they could reach all organs through blood, stimulate the body to produce a series of inflammatory cytokines, and eventually lead to cytotoxicity, which increases the risk of cardiovascular events (Suwa et al., 2002).

Nanomaterials have a large number of surface atoms and are highly reactive, which can generate free radicals and stimulate the formation of ROS, thereby interfering with antioxidant systems (Chen and Von, 2005). Oxidative stress can induce oxidative damage to macromolecular substances, such as DNA and proteins, which leads to cell growth inhibition, cell cycle abnormalities, and cell death.

The study on the toxic mechanism of cardiovascular system damage caused by nanomaterials in global is still in its infancy. There is very few relevant research evidence on the biological endpoints to determine the relationship between the physicochemical parameters (shape, size, size distribution, surface structure, electrochemical properties, etc.) of the nanoparticles and the toxic effects of the cardiovascular system. Therefore, scientists need to carry out more researches on the cardiovascular system toxic effects and mechanisms of typical nanomaterial exposure, which can make better use of the positive effects of nanomaterials to prevent, reduce or eliminate the possible adverse effects on health. Furthermore, it would provide theoretical and technical basis for the establishment of nanomaterial safety evaluation technology and standards.

## Summary and Perspective

In conclusion, the nano-carrier, as an efficient, specific and controllable intracellular drug delivery method, has shown unique advantages in the diagnosis and therapy of CVDs. It can effectively solve the problems of targeting, local drug delivery, controlled release, sustained release, and reducing toxicity while it is developing toward the multifunctional and integrated direction of diagnosis and therapy. With the innovation of nanotechnology and the deepening studies on molecular pathological mechanism of CVDs, the application of NDDSs will be promoted, and new techniques and methods will be provided for clinical diagnosis and therapy. In addition, since the study on these nano-carriers is in its infancy, many problems still remain unclear. The main challenge is how to solve the biocompatibility of nano-drug-loaded particles themselves or their degradation products, which is need to be solved in the field of nano-biomedicine in the future.

## Author Contributions

WL proposed and developed the research outline, MY contributed to the concept and content framework, YD wrote the first draft and XZ prepared the drawings and contributed to improving the draft. HS and QH polished the article. ZW modified the format.

### Conflict of Interest

The authors declare that the research was conducted in the absence of any commercial or financial relationships that could be construed as a potential conflict of interest.
